# Ankylostomiasis or Uncinariasis1Read in the Section on Stomatology of the American Medical Association, at the fifty-sixth annual session, July, 1905, and republished with request of the Section, through the courtesy of the Journal of the American Medical Association.

**Published:** 1905-12

**Authors:** T. M. Russell Leonard

**Affiliations:** L.R.C.P., L.R.C.S. (Ed.), Grenada, British West Indies


### ANKYLOSTOMIASIS OR UNCINARIASIS.[1]

T. M. RUSSELL LEONARD, L.R.C.P., L.R.C.S.(ED.), GRENADA, BRITISH WEST INDIES.

THIS disease, an endemic anæmia caused by the parasite, *Ankylostoma duodenale* or *Uncinaria duodenalis* and *Uncinaria americana* (Stiles), is a dangerous one both to the individual and to the community in general, leading as it does in individual cases to degeneration of important organs, resulting probably in death and to widespread infection and deterioration among the community in general.

The parasite is now too well known to need a full description; in Grenada, B. W. I., the large majority of cases show the old world parasite,—viz., *A. duodenale,* but in many cases I have come across the variety described and named by Stiles, *Ankylostoma* or *Uncinaria americana,* the new world variety. The chief differences between these two species lie in the size and the armature of the mouth, the *Ankylostoma duodenale* being larger, with a heavily armed mouth,—viz., two pairs of hook-like ventral teeth and one pair of dorsal,—the *Uncinaria americana* being smaller, with two pairs of ventral plates or lips and one dorsal pair (Stiles). Microscopically, the worms appear as short threads white or gray in color, according to the contents of the genital organs; usually I have found that the white are males and the gray colored ones females with ova; I have only occasionally seen them blood-red.

---

[1] Read in the Section on Stomatology of the American Medical Association, at the fifty-sixth annual session, July, 1905, and republished with request of the Section, through the courtesy of the Journal of the American Medical Association.

### DEVELOPMENT.

The ova develop in earth under favorable conditions of humidity, temperature, and shade. I have found that a certain proportion of water is necessary. In undiluted fæces the larvæ appear but soon die; in fæces diluted with much water, in a few cases of mine the larvæ appeared, but did not live; but in the majority the ova did not develop. The ova developed at the ordinary temperature in this island,—viz., 80° to 90° F.,—but the larvæ are killed and the ova prevented from developing when placed in ice. Direct exposure to the sunlight killed the larvæ when these had developed, and also prevented the ova from developing, so that a certain amount of shade is required for the development of the larvæ from the ova. Each ovum only produces a single larva, and this never takes place within the host, but outside, so that an increase of adult worms in any particular case is impossible without reinfection from without; this point is therefore of great importance both in the treatment and in the prevention of this disease.

### MODES OF INFECTION.

Two methods of infection are possible: 1. By the ingestion of the larvæ in the food or water. 2. By penetration through the skin and subsequent migration to the intestine, where the embryos mature into the adult worms.

In Grenada the class found to be chiefly affected with the disease are the laborers and agriculturists, consisting chiefly of negroes, East Indians, and poor whites. The huts occupied by them are small, raised a few feet off the ground in most cases, entirely surrounded with vegetation almost up to the walls, in this island being chiefly bananas and cocoa and other trees planted to protect the growing cocoa. Sanitation is unknown among them, and latrines are never used, the fæces being deposited on the earth among the trees, so that in time the soil around these huts and also in the fields where they work becomes heavily infected. During the rains the mobile larvæ, also ova, are washed down into the pools, shallow wells, and streams, and as most of the villages depend on such for their drinking water, it can be readily understood that infection in this manner—viz., by ingestion—can take place; helped to a very great extent by the dirty habits of the people, such as eating with soil-stained hands, improperly washed vegetables and fruit, and, as above stated, drinking water infected with the larvæ and contained a large quantity of earthy matter in suspension. I am of opinion that in this island at any rate this method of infection takes place, as the drinking-water contains to my knowledge both the living larvæ and earthy matter in suspension, so that although many observers have remarked, and I have proved to myself, that the embryos die on drying and that the earth taken in must be moist, yet in the above condition we have both earth and moisture. True geophagy is not practised to any extent in this island, chiefly among children, so that a few cases may be set down as due to this method of infection.

The second method of infection—viz., penetration of the skin by the larvæ—is without any doubt the true one, as proved by the experiments of Looss and others. In every case treated by me I have elicited the fact that the patient has suffered from a form of dermatitis known in this island as "chauffie" or "ground-itch" prior to his anæmia, the period ranging from three months to a year or two, and in advanced cases they have had attacks every year, especially during the rains. I have had ten cases of this dermatitis under treatment in the hospital, and the following were the chief characteristics: in cases of recent infection scattered vesicles were seen, many of which broke down, leaving small ulcers, usually affecting the lateral side of the feet and the urb of the toes; in older cases the ulceration was more extensive, deep cup-shaped ulcers being seen on the dorsum and lateral parts of the foot and between the toes. Some of these cases resisted treatment for some time, although in the early cases they healed quickly. These cases remained under treatment, and in about three to six months from the date of the first appearance of this dermatitis they complained of uneasiness in the epigastrium, in some cases actual pain and other dyspeptic symptoms, and on my examining the fæces later I found the characteristic ova, thus showing that this dermatitis known here as "chauffie" is caused by the penetration of the skin by these larvæ. This skin infection is widespread in the island, especially on the estates and among the villages, and nearly every adult laborer has had it, and also the children who are allowed to roll about and play in the earth around the huts.

The disease chiefly affects the country, and very few cases are seen in the larger towns, and this fact is easily explained as follows: No sanitation in the villages; fæces deposited on the earth among the bananas and cocoa trees; the ideal temperature, shade, humidity, and decaying matter being afforded by these groves for the development of the ova, and the poverty and filthy habits of the class as a whole, necessitating walking barefooted, infecting himself and then carrying it to his hut and infecting the children in turn. With regard to immunity from this disease, there is none; all classes may become infected, but the poorer classes are more liable not only from the nature of their work and poverty, but also from the inadequate diet both in quality and quantity.

### SYMPTOMATOLOGY.

Within the past year, April, 1904, to April, 1905, I have had two hundred and fifteen cases under my treatment and observation at the Colony Hospital. These cases were simply admitted as they applied, and a large number also suffered from other complications. I found that uncinariasis could be divided into two distinct classes, (*a*) acute and (*b*) chronic, the acute class being further subdivided into (1) mild cases and (2) moderate. On a microscopic examination of the fæces the ova can be readily detected, but the number present in the field is no guide to the extent of the infection in many cases, although if large numbers are seen it is probable there are a large number of parasites, but the reverse is not necessarily correct. The mere presence of this parasite does not constitute the disease, and these early cases are only of importance from their being a source of danger to others and to the district or village in which they reside.

*Mild Cases.*—Pain or uneasiness in the epigastrium often relieved, but usually increased, by the ingestion of food. Appetite lost but in many cases ravenous. Frontal headache, dizziness and palpitation, breathlessness on exertion, and indisposition to do any work, either physical or mental. In these cases anæmia was not marked and pallor was only slight, being yellowish in whites and muddy in the colored races.

*Moderate Cases.*—In these cases all the above symptoms were pronounced; pallor is more marked and the anæmia prominent. The mucous membranes and conjunctivæ pale, skin dry, appetite in the majority of cases lost, nausea and vomiting present in many cases, pain in the epigastrium. Headache is very severe and continued, referred chiefly to the frontal region, often temporal, vertigo and extreme tinnitus present. Palpitation is severe, and breathlessness ensued on the slightest exertion. Great languor and lassitude is complained of, and fever of an irregular, intermittent type is present with pains in the joints.

*Chronic or Very Advanced Cases.*—Here the anæmia is intense and pallor very extreme, the patient having an icteric tinge of the sclera. Œdema of the feet and ankles is present, with general anasarca in the majority of cases, and in eight cases under treatment ascites was present. The digestive symptoms have changed, either becoming more pronounced, with vomiting or ingestion of food, or in many cases they disappear altogether. Precordial pain and pain in the chest is complained of, palpitation is severe, and dyspnœa comes on with the slightest exertion; the pulse is weak and compressible, and pulsation of the large veins of the neck is noticed. Vertigo and tinnitus is extreme, this latter symptom often causing sleeplessness. Dimness of sight is complained of, and in many cases the pupils are noticed to be dilated. The urine is normal in quantity, usually pale in color, and in only three cases was albumin present. On auscultation, bruits are heard over the veins, and there is a well-marked murmur on the valve areas, and the heart itself is often dilated, with the apex-beat depressed. The muscular system is weak, although the muscles appear plump and well nourished and the patient is absolutely incapacited from any kind of work. The patient is listless and apathetic and the temperature is often raised, but in the majority of cases it is subnormal.

*Digestive System: Stomatitis.*—In two cases only have I seen a stomatitis affecting the tongue and mucous membrane of the mouth, and both these cases were in the advanced stages. I do not attach any importance to it, as I have not seen it present in other cases. Pyorrhœa alveolaris is very common in this island among the laboring class, also caries of the teeth, and may be noticed in all the cases of the disease, but I do not see any connection between them.

*Tongue.*—In the acute and chronic cases the tongue is very pale, almost white in color, usually coated but often red and rawlooking and denuded of epithelium. I have remarked a peculiar pigmentation seen in every case except the poor whites. It consists of a black or blue-black pigmentation, in minute spots, either singly or in patches, usually on the tip and sides of the tongue; it is not seen, as I mention above, in cases in whites, but only in the pigmented races, negroes and East Indians. I am of opinion that there is some connection between this pigmentation and the disease, but so far I have not been able to establish it. The digestive symptoms are very similar to those due to dyspepsia, and often in the mild cases are simply treated as such, the fæces not being examined, with the result that the case steadily progresses instead of improving. Appetite is variable, being lost in many cases, but ravenous in others; pain or uneasiness in the epigastrium is a well-marked symptom, and is likened by the patient to the sensation of having a stone in the stomach; this symptom is usually increased by the food taken by the laboring class here, which is of poor quality and very coarse and bulky, quantity instead of quality being preferred. Flatulence and palpitation is very common, and the bowels are usually constipated; only in the very advanced cases have I seen diarrhœa.

*Circulatory System.*—On inspection, the apex-beat is found to be pronounced, especially if the patient is made to walk a little, and is usually displaced downward and to the left, but in many of the chronic cases it is feeble. On percussion, the cardiac area is found to be enlarged in the second class of cases, and in the chronic cases dilatation is present. On auscultation, bruits are heard, very marked in the third left space, and in eight cases under my treatment they were practically organic, fatty degeneration and dilatation having occurred. (Two of these cases died in hospital and enabled me to make an autopsy.) Precordial pain and pain in the chest, palpitation, and dyspnœa are present and are very distressing symptoms in chronic cases. Vertigo and tinnitus aurium are also other well-marked symptoms, especially in the later stages, and are no doubt adjuncts in causing the inability of the patient to get about and in producing the sleeplessness complained of in many cases.

*Nervous System.*—Symptoms pointing to this system are languor and lassitude, mental apathy, insomnia, and the severe and continued headache, usually frontal, often temporal, and sometimes the pain is referred to the whole head. Numbness of the feet and formication are complained of as well.

*Integumentary System.*—The skin is harsh and dry, and in the chronic cases general pruritus is often a constant and very annoying symptom; atrophy is present in chronic cases.

*Fæces.*—In all the above cases I have not seen any blood in the stools, but in six cases I have noticed blood-stained mucus, although it is not a constant symptom. I have mentioned that constipation is usually complained of, but diarrhœa may occur. Microscopically the ova are readily seen, in conjunction with those of *Ascaris lumbricoides* and *Tricocephalus dispar,* but I have also found them in conjunction with those of *Oxyuris vermicularis,* and in twenty cases *Rhabdonema intestinalis* was present.

*Blood Examination.*—On a microscopic examination the point that is noticed is an increase of the eosinophiles, but on making a count there is no marked leucocytosis; the percentage of hæmoglobin varied, but there was a slow but steady decrease as the disease advanced; it was also noticeable that after treatment and when the patient began to improve the red corpuscles increased, but the percentage of hæmoglobin often fell before it began to rise.

The following table shows the percentage obtained by me from blood-counts in twenty cases: Eosinophiles, 18; polymorphonuclear, 55; large lymphocytes, 8; small lymphocytes, 17; other cells, 2; total, 100. Hæmoglobin varied from eight to forty per cent.

*Diagnosis.*—The diagnosis of the disease depends not so much on the symptoms, which, although they may be well known, are very often mistaken for those of dyspeptic troubles in the mild cases and of heart disease in the advanced cases, but on the discovery of the ova in the fæces. The ova are very characteristic and cannot be mistaken for those of any other parasite, as they are unstained by bile, while those of the other parasites are usually stained yellow. The ova of the *Oxyuris vermicularis* may be mistaken by beginners for that of *Ankylostoma,* but a little practice enables them to be easily distinguished. The *Ankylostomum* ova are perfectly clear and chitinous, shelled with a granular grayish vitellum, usually segmented into four, sometimes six or eight, parts; those of *Oxyuris* are smaller and the vitellum is not segmented, but fills up the entire shell. In many cases the temperature is of an irregular intermittent or remittent type, patients complaining that the fever comes on every night or morning, and this fever often causes the disease to be mistaken for malaria. A microscopic examination of the blood soon clears this point, although malaria is very often, especially in certain districts of this island, a complication of the disease, and when the case is treated as one of malaria, without an examination of the fæces having been made, the practitioner is surprised to find that quinine has, seemingly, no effect on the temperature. (I had a case of this nature sent into hospital—*vide* report on case attached.)

### MORBID ANATOMY.

The following data are taken from four autopsies made by me at the hospital on bodies of patients who died from advanced uncinariasis. One died suddenly (forty-eight hours) on the second day after admission (this case I have described separately, with a complete history).

*General Condition.*—The bodies appeared plump and well nourished, the skin was harsh and dry, of a dirty, muddy color, œdema of the feet and ankles with ascites was present, and there was serous effusion into the various cavities,—viz., pericardium, and ventricles of the brain; subcutaneous fat was abundant and yellow in color.

*Heart.*—The heart was found to be enlarged, fatty degeneration and dilatation being present with incompetence of the valves; the heart walls were thin and flabby, and patches of atheroma were present in the aorta. The cavities were found filled with soft dark clots.

*Lungs.*—The lungs appeared normal, although pale in color, but in one case advanced pulmonary tuberculosis was present and was no doubt the cause of death. Pleural cavities contained about a pint of clear, yellowish fluid.

*Spleen.*—This organ appeared normal in two cases, but in the others was enlarged and fibrous, probably due to malaria, as both cases came from a very malarious district.

*Liver.*—This was pale and slightly enlarged in all the cases; fatty degeneration was present and the tissue very friable.

*Kidneys.*—In one case the kidneys were small, pale, capsule adherent (chronic interstitial nephritis), but in the others they appeared normal although pale in hue.

*Stomach.*—This organ showed signs of chronic catarrh, in two cases dilatation was present, but I did not find any uncinaria in the cavity of the organ.

*Intestines.*—The parasite was found in large numbers, chiefly in the upper part of the jejunum, but also in the duodenum. The parasites were lying free in the lumen of the bowel, but a great many were attached to the intestinal wall. There was a large quantity of mucus present in the intestine, in which the worms were lying embedded. The mucous membrane should show minute hemorrhagic spots, as well as pigmented spots. In one case the wall itself was very thin. There was no blood in the intestines.

*Brain.*—The brain showed anæmia, with effusion into the ventricles of a pale yellowish fluid.

The above four cases were the only ones that died and enabled me to make an autopsy. The majority of the cases made rapid and uneventful recoveries, but a few of the very advanced cases were slow, and remained in hospital over three months before being discharged.

### PIGMENTATION OF TONGUE AND ITS PROBABLE CAUSATION.

In every case of ankylostomiasis under my treatment, with the exception of the poor whites who are of European parentage, the pigmentation of the tongue is seen. It consists of minute black, blue-black, or brown spots, usually on the lip and sides of the tongue; sometimes the pigmentation is seen in larger areas. The colored races in this island—viz., the negro, East Indian, and their descendants—show this pigmentation in every case of uncinariasis, and it is especially marked in the chronic cases.

Pigmentation of the skin and mucous membranes may be derived either from the blood or bile or from external agents. In these spots the pigment is deposited chiefly in the mucosa of the papillæ in its lower layers, but also in the upper layers. There is no doubt that disturbed innervation plays a part in producing this result, as is seen in the bronzing of the skin in Addison's disease, which is probably due to inflammation of the abdominal sympathetic. The pigment melanin closely resembles hæmatin, one of the constituents of the hæmoglobin, in its composition, hence we can conclude that it is derived from that source. Its presence is usually associated with two very opposite conditions of nutrition, being sometimes an accompaniment of tissue degeneration and at others of active trophic changes. It is well known that the chlorophyl of plants and the hæmoglobin of the blood are intimately associated with respiratory changes. In the majority of cases where pigment is found some concomitant blood change is also present, as, for instance, in malaria, pigment is formed from the destruction of the red corpuscles and deposited in various organs of the body, and without doubt deposition of pigment is under the control of the sympathetic system.

In uncinariasis we have a toxin produced by the parasite and which is the true cause of the profound anæmia, blood and nervous changes. Here, therefore, we have both the necessary factors for the production of pigmentation, not only on the tongue and mucous membranes, but elsewhere,—viz., (1) blood changes in which the hæmoglobin of the red corpuscles is broken and disintegrated, as shown in the reduction of the hæmoglobin percentage as the case advances, and (2) disturbance of the nervous system by the same toxin which affects all centres, sympathetic included, as evidenced by the symptoms.

Dr. Laycock has observed that pigmentation may be regarded as having reference to the excretion of carbon after it has served its purpose in the tissues, and hence, pathologically, pigmentation may be taken to be expression of (1) imperfect oxidation of the carbon, so that it is not eliminated as carbonic acid from the body; (2) excessive production of carbon from highly carbonaceous food; (3) pigment resulting from the disintegration of the hæmoglobin of the red cells. In uncinariasis all three of these factors are present, produced by the toxin excreted by the parasites,—(1) diminished oxygenating power of the blood from destruction of the blood-cells, so that carbon waste is not got rid of; (2) presence of hæmatin from disintegration of the hæmoglobin; and in this island (3) we have the third factor,—viz., excess of carbonaceous food, as the laborers all live on coarse, bulky food, the quantity rather than the quality being the chief item of importance. The class of people in whom the disease is chiefly seen are the colored races, and, therefore, more liable to pigmentation than those races in whom pigment is not usually present in the skin. From the above facts I am of opinion that this pigmentation of the tongue and buccal mucous membranes and mucous membrane of the intestine has some connection with the disease, and may be taken as of diagnostic value, as I have never failed to find the ova and the other symptoms of the disease present in any case which showed this pigmentation.

Another view of its causation may be taken in the dermatitis or "chauffie" affecting the laboring class in this island and which without doubt is the true method of infection. Pigmentation of the skin affected takes place in this island, where the class affected are dirty in their habits and careless in regard to eating unwashed fruit and vegetables. Muddy drinking-water is one method of infection, as during the rains the water supply, which usuualy consists of shallow wells and ponds, is contaminated to a large extent, and as the larvæ do not take very long to penetrate the skin, as seen from Claude Smith's experiments, it may be possible that, when taken into the mouth, they make their way more easily and quickly into the mucous membrane, and that these pigmented areas are the points of their entrance.

Case I.—A. J., aged nineteen, unmarried, living with his parents, laborer in cocoa estate. Admitted to Colony Hospital, February 2, 1905.

*History.*—Patient has been ill for the past year with general weakness, as he termed it, unable to do much work, owing to breathlessness and palpitation. Three weeks prior to admission he had a severe rigor, followed by fever and slight perspiration; fever comes on every day. Has lost flesh and is emaciated. Complexion muddy; lips and buccal mucous membranes very pale; tongue shows minute pigmented spots on lip and sides. Conjunctivæ very pale, and scleræ have an icteric tinge; appetite very poor, can only take fluid nourishment; pain and tenderness in epigastrium; nausea and vomiting after food; bowels constipated and have usually been so for the past year. Skin harsh and dry, and does not perspire very much; itching sensation over the whole body. Precorial pain and pain in the chest complained of; palpitation marked, with dyspnœa in exertion. Dimness of sight, with severe and continued frontal headache and tinnitus aurium. Vertigo and sleeplessness present. Very weak, and has not left his bed for the past three weeks. Pulsation of veins in the neck; apex-beat depressed and to the left; marked bruit in third left intercostal space. Pulse soft and compressible.

*Examination of Fæces.*—Ova of uncinaria present in large numbers with those of *Ascaris lumbricoides* and *Tricocephalus dispar.* No blood in the stools.

*Examination of Blood.*—Malaria Æstivo-autumnal parasite present. Eosinophiles, 20; polymorphonuclear, 58; large lymphocytes, 10; small lymphocytes, 12.

Temperature on admission, 102.6°, rising on the day after to 104°. No marked rigor or diaphoresis.

*Diagnosis.*—Ankylostomiasis or uncinariasis. Complication, malignant malarial fever.

*Treatment.*—Saline aperient every morning. Hypodermic injection of quinine bihydrochlor. grs. 5 every morning at ten and in the evening. Diet: Milk, beef tea, arrowroot, etc.

*Course.*—Temperature still continued. February 17, blood re-examined and found free of malarial parasites. Temperature, 99.8°. Fæces contained the ova in numbers. February 20, 120 grs. thymol given in cachets of 40 grs., followed by a saline aperient four hours after the last dose; stools carefully washed and found to contain large numbers of worms. Temperature reduced, but still above normal. Thymol repeated February 23 and stools examined, worms still being passed.

Repeated thymol February 26 and 28, and after the last dose only a few worms (six) were found in the last stool.

Fæces examined March 2; no ova present, but thymol repeated once more, and a week later they were again examined, but no ova present; similarly on the 17th.

The diet was now changed, as appetite had increased; after the second dose of thymol the headache disappeared and the tinnitus was very much decreased, and patient slept well at night. He was now put on the following mixture as well:

| | | | |
|---|---|---|---|
| ℞. | Tinct. ferri perchlor | ʒiiss | 10 |
| | Liq. arsenitis hydrochlor | ʒi | 4 |
| | Mag. sulph | ʒiv | 15 |
| | Syrupi aurantii | ʒiss | 6 |
| | Aquæ, q. s. ad | ℥viii | 240 |

M. Sig.—One tablespoonful three times a day after meals.

Blood examination, March 31: Eosinophiles, 13; polymorphonuclear, 65; large lymphocytes, 10; small lymphocytes, 7. Hæmoglobin percentage, 22.

Blood examination, aMrch 31: Eosinophiles, 13; polymorphonuclear, 45.5; large lymphocytes, 14.8; small lymphocytes, 26.2.

Patient is doing well, has gained weight and flesh, all symptoms have disappeared, skin moist. Rapid and uneventful convalescence.

Case H.—R. G., aged thirty-six, married, children, laborer.

*History.*—Patient has been ill for past six years, gradually getting worse each year; weakness, fever, breathlessness, and palpitation; disinclination or inability to work. Husband and children also affected similarly. Has had "chauffie" very often, particularly after or during the rains. Appearance plump and well nourished. Very anæmic; has been very ill for the past month; bedridden; brought to hospital in a chair.

*Examination.*—General anasarca very marked; ascites developed; face and scalp puffy and œdematous. Says she never perspires; appetite lost; pain and tenderness in epigastrium; vomiting after food. Tongue very pale and white; teeth indentations on tongue; numerous minute pigmented spots on lips and sides, with bluish streaks on side as well.

Superficial veins of abdomen distended; diarrhœa; fæces yellowish-brown, no blood; on examination ova of ankylostoma and ascarides were seen. Liver enlarged; abdomen tender on pressure; ascites present. Precordial pain and pain in the chest very severe; dyspnœa extreme, cough troublesome, bases of lungs dull on percussion.

Extreme vertigo; marked tinnitus aurium, with severe continued frontal headache; sleeplessness, and has had fainting attacks.

Vision dim, great weakness. Heart markedly dilated, with bruits, pulsation of veins in neck; apex-beat full and diffused. Pulse 110, weak and dicrotic. Urine normal; patient quite sensible, but prefers being left alone. Temperature on admission 97.8°.

*Diagnosis.*—Ankylostomiasis, chronic.

*Treatment.*—Milk and beef tea, stimulants.

Patient did not do at all well, and died suddenly on the second day after admission.

*Blood Examination.*—Eosinophiles, 8; polymorphonuclears, 56; large lymphocytes, 6; small lymphocytes, 30. Hæmoglobin, 10 per cent.

*Morbid Anatomy.*—Lungs very pale, but otherwise normal; bases œdematous, and pleural cavities contained about two pints of straw-colored fluid.

Heart: Pericardium contained four ounces of serous fluid. Heart very much enlarged and dilated; cavities filled with soft, dark clots. Fatty degeneration present in the walls; valves inefficient.

Liver enlarged, pale, and fatty degeneration present. Abdomen contained large quantity of ascitic fluid.

Spleen slightly enlarged, pale, but otherwise normal.

Intestines: Jejunum and duodenum both contained large quantities of the worms, most of which had dropped and were lying in the lumen of the bowel. Mucus present in large quantities. Large intestine contained dark yellowish-brown fluid fæces. Intestinal wall thin and atrophied in parts, presenting large numbers of minute hemorrhagic and pigmented spots.

Stomach: Signs of chronic catarrh present; no worms in the cavity itself.

Kidneys pale and otherwise normal.

Brain anæmic and ventricles filled with fluid. Pia mater stripped easily off the cortex, serous fluid beneath it filling the sulci.

Death in this case was due to mitral inefficiency, resulting from dilatation of the heart caused by chronic uncinariasis or ankylostomiasis.

### TREATMENT.

Thymol is the drug employed by me, and I have had the very best results, with no single ill effect, even in children. The dose given by me always varies with the age of the patient and also his general condition; it ranges from ten grains to a child to forty grains for an adult at a single dose.

The following is the procedure adopted by me at the Colony Hospital: The patient is carefully prepared for three or four days after admission, the bowels are kept active by saline aperients administered every morning in sufficient dose to cause one evacuation, the diet is light and easily digested,—viz., milk, beef-tea, arrowroot, etc., with pepsin if required. In very advanced cases with cardiac troubles, stimulants and cardiac tonics administered. The chief object in the administration of the aperient being to get rid of the mucus with which the intestine is often loaded and in which the parasites lie buried.

At ten P.M. on the night prior to the administration of the thymol a dose of saline aperient is given, and no food except a pint of milk given to the patient next morning, and two hours afterwards the first dose of thymol is given. I give it in the form of cachets each containing five grains for a child and forty grains for an adult, and one cachet is taken every hour till fifteen grains are given to the child and one hundred and twenty grains to the adult. In old people and very advanced cases in adults I usually give thirty grains instead of forty grains. No food is allowed the patient, and four hours after the last cachet is given a saline aperient is given and the patient allowed his diet. The stools are collected and carefully washed, and invariably show a large quantity of worms. In the case (1) described by me five hundred worms were passed after the first administration of thymol. The above treatment is carried out on three alternate days, occasionally an interval of four or five days is allowed in debilitated cases; the fæces are carefully examined for ova a week afterwards and usually found free. The fæces are again examined two days before the patient is discharged, and it is my invariable practice to give another single dose of thymol and to carefully examine the stools again. The after-treatment of the case consists in the administration of tonics, chiefly iron and arsenic, often combined with strychnine; the diet is increased, but is still one which is easily digested and nourishing, and usually the patients seek their discharge in from a fortnight to three weeks after the fæces are free of ova; and are recommended to continue the tonic at their own homes for a still longer period. I usually administer the iron and arsenic in the form mentioned in the report of Case I above, as I find it gives the best results in a shorter period of time.

The saline aperient used by me, and which is preferable to any other aperient, is the following:

| | | | |
|---|---|---|---|
| ℞. | Sodii sulphas, | | |
| | Mag. sulphas | āā ʒiss | 6 |
| | Aqua | ad ℥ii | 00 |
| M. | Sig.—Drink. | | |

As I have previously stated, I have had no ill effects from administering thymol, although a few cases have complained of dizziness and a sensation of fainting, but it has gradually passed off; it is, as we know, a very insoluble drug except in spirits and oils, and therefore is not absorbed in sufficient quantity to cause toxic effects, besides which the saline purge given shortly after the last dose hurries it out of the system, although sufficient time has been given the thymol to act on the parasites, and besides this there is no doubt that sodium or magnesium appears to be an antidote.

A remarkable fact, and one which confirms me in the use of thymol, is the immediate relief given by the first administration of the drug from the severe headache and other mental symptoms, the patient next day feeling much brighter and better. Bentley recommends and prefers betanaphthol to thymol. I have not tried this drug as yet, being so far satisfied with my results from thymol, but I intend giving it a trial and comparing the results.

### PROPHYLAXIS.

In this island the two chief factors in the causation of this disease are (1) muddy and infected drinking-water; (2) soil pollution and infection through the skin. With regard to the first, it has been doubted whether this factor is a true one, yet I have had two cases of this disease in which there was no history of dermatitis, the patients being fairly well to do and not exposed to the second method of infection, but they admitted that the first cause might be the real one, as the water supply was obtained from shallow wells, liable to infection, and, especially during the rains, was often muddy.

However, there are only three methods of prevention,—viz., 1, prevention of water infection; 2, prevention of soil infection; 3, treatment of all cases of the disease.

The last two are the chief ones. In this island the disease is widespread throughout all the districts, especially on the estates and in certain villages, hence the chief essential is the prevention of the fecal contamination of the soil in order to eradicate the disease; the promiscuous depositing of fæces near the huts and villages and in the fields should be prevented, but owing to habit and the low intelligence of this class it seems an impossibility.

On all estates where huts are provided for the laborers and other families easily accessible privy accommodation should be provided; pits and trenches would do, which could be filled in from time to time and fresh ones opened. Another point is to try to destroy all ova and larvæ on heavily infected fields by ploughing the earth over and burning bush fires over it and replanting, but as most of the cultivation here is cocoa this is an impossibility; these cocoa groves are the chief infected areas, as here the ova have the ideal humidity, shade, and temperature for their growth and development. The people should, therefore, be made acquainted with the danger of depositing fæces promiscuously and thus infecting the soil and the liability of their acquiring the disease owing to the nature of their work, barefooted. The next point is the proper examination and treatment of all cases of the disease. On all estates and in villages an examination of all cases of dyspeptic troubles, dermatitis, and anæmia should be made and the fæces examined, and, if the ova are found, immediate treatment of the case begun, so as to prevent their becoming a source of danger to others who are not infected. This cannot be done without an outlay on the part of the estate owner or the government itself, but there is no doubt that to thoroughly eradicate this disease such measures must be adopted; otherwise the treatment of cases individually is of little use, for as soon as they are cured and go back to their work, they are reinfected.

### DISCUSSION.

Dr. V. A. Latham, Chicago, said that the southern parts of this country were now confronted with this condition. In patients with ankylostomiasis the whole system stops developing; they are anæmic, frail, and almost infantile in their actions and characteristics.

Dr. Eugene Talbot, Chicago, said that two or three similar cases have occurred in this country, but not enough to warrant the conclusions brought out by Dr. Leonard. One occurred lately in Southern Illinois.

